# Effect of Tai Chi on Cognitive Function among Older Adults with Cognitive Impairment: A Systematic Review and Meta-Analysis

**DOI:** 10.1155/2021/6679153

**Published:** 2021-08-05

**Authors:** Renjun Gu, Yujia Gao, Chunbing Zhang, Xiaojuan Liu, Zhiguang Sun

**Affiliations:** ^1^Jiangsu Provincial Second Chinese Medicine Hospital, The Second Affiliated Hospital of Nanjing University of Chinese Medicine, Nanjing 210017, China; ^2^Rehabilitation Medical Center, Jiangsu Province Hospital, Nanjing 210029, Jiangsu, China; ^3^Jiangsu Hospital of Chinese Medicine, Nanjing 210046, Jiangsu, China; ^4^The First School of Clinical Medicine, Nanjing University of Chinese Medicine, Nanjing 210023, Jiangsu, China

## Abstract

**Background:**

Cognitive decline occurs in all persons during the aging process and drugs can only alleviate symptoms and are expensive. Some researches demonstrated that Tai Chi had potential in preventing cognitive decline while others' results showed Tai Chi had no influence on cognitive impairment. Therefore, we conduct a systematic review and meta-analysis to assess the efficacy and safety of cognitive impairment patients practicing Tai Chi.

**Methods:**

A comprehensive literature search was carried out in multiple databases, including PubMed, Cochrane, MEDLINE (Ovid), Web of Science, Embase, Scopus, PsycInfo (Ovid), CKNI, Wan Fang, VIP, SinoMed, and ClinicalTrails, from their inception to 1 July 2020 to collect randomized controlled trials about practicing Tai Chi for patients with cognitive impairment. Primary outcomes included changes of cognitive function and secondary outcomes included changes of memory functions. Data were extracted by two independent individuals and Cochrane Risk of Bias tool version 2.0 was applied for the included studies. Systematic review and meta-analysis were performed by RevMan 5.3 software.

**Results:**

The results included 827 cases in 9 studies, of which 375 were in the experimental group and 452 were in the control group. Meta-analysis showed that Mini-Mental State Examination WMD = 1.52, 95% CI [0.90, 2.14]; Montreal Cognitive Assessment WMD = 3.5, 95% CI [0.76, 6.24]; Clinical Dementia Rating WMD = −0.55, 95% CI [−0.80, −0.29]; logical memory delayed recall WMD = 1.1, 95% CI [0.04, 2.16]; digit span forward WMD = 0.53, 95% CI [−0.65, 1.71]; and digit span backward WMD = −0.1, 95% CI [−0.38, 0.19]. No adverse events were reported in the included articles.

**Conclusion:**

There is limited evidence to support that practicing Tai Chi is effective for older adults with cognitive impairment. Tai Chi seems to be a safe exercise, which can bring better changes in cognitive function score.

## 1. Introduction

While social aging is a trend, cognitive decline can occur for everyone. Eventually, this may result in mild cognitive impairment and dementia [1]. Mild cognitive impairment occurs along a continuum from normal cognition to dementia [2]. It is widely recognized as the intermediate stage of cognitive impairment between the changes seen in normal cognitive aging and dementia [3]. At present, drugs for cognitive impairment can only alleviate the symptoms of cognitive disorders and their price is usually high. Therefore, complementary and alternative therapies have become a hot research topic for improving cognitive impairment in recent years [4].

Tai Chi has a long history and culture. Participants take deep breathing and mental concentration in order to carrying out smooth and continuous body movements [5]. It combines Chinese martial arts and meditative movements that promote balance of mind and body for healing [6]. Physical exercise and fitness have been proposed as potential factors that may promote healthy cognitive aging [7] and aerobic exercise was proven to improve cognitive function in adults with neurological disorders [8]. In recent years, long-term cognitive training and physical exercise had been confirmed its benefits for delaying the cognitive decline for the elderly [9, 10]. Tai Chi might improve memory and executive function in older adults with amnestic-mild cognitive impairment, possibly via an upregulation of brain-derived neurotrophic factor [11, 12]. The studies suggested Tai Chi has impacts on global cognitive functions, visuospatial skills, semantic memory, verbal learning memory, and self-perception of memory [13]. It may also have direct benefits on enhancing attention and executive functions [14].

At present, a growing body of evidence supports that Tai Chi may help improve cognitive function and mental well-being for older adults with mild dementia [15, 16]. It also proved that Tai Chi has psychophysiological benefits for motor coordination and memory [17–20]. However, some studies reported no significant differences in assessment of cognitive function [21]. Previous meta-analysis revealed that Tai Chi had no influence on individuals with cognitive impairment [22]. In addition, previous meta-analysis only searched the English databases [23] while Tai Chi is most practiced in China. Therefore, we will conduct a meta-analysis and systematic review without language limitation to assess the effect of Tai Chi on cognitive function among older adults with cognitive impairment.

## 2. Information and Methods

### 2.1. Study Protocol

This systematic review and meta-analysis followed the preferred reporting items for systematic reviews and meta-analyses (PRISMA) of 2015 guideline [24]. The protocol was registered at PROSPERO (http://www.crd.york.ac.uk/PROSPERO), registration number: CRD42020171559.

### 2.2. Search Strategy

**Figure 1:**
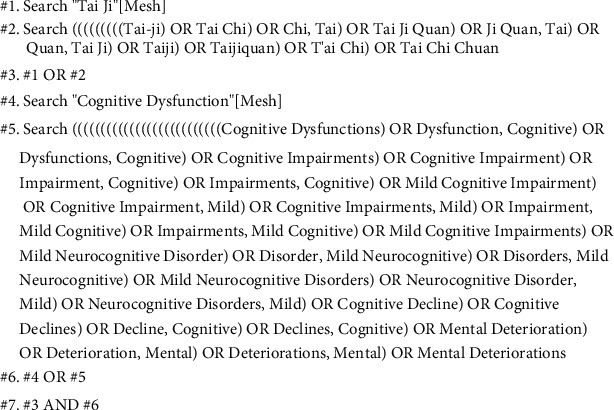
Literature search strategy.

Electronic literature searches were performed in the database of PubMed, Cochrane, MEDLINE (Ovid), Web of Science, Embase, Scopus, PsycInfo (Ovid), CKNI, Wan Fang, VIP, SinoMed, and ClinicalTrails from inception to July 2020. Search strategy of PubMed is shown in Figure 1.

### 2.3. Inclusion Criteria

(a) Published literature; (b) RCTs; (c) inclusion of people with cognitive impairment; (d) people over 65 years old according to classification for older adults by World Health Organization in 2020 [25]; (e) practicing Tai Chi for more than one month but no more than one year; (f) interventions using Tai Chi as a main treatment; the combination therapy of Tai Chi and other interventions compared with the same other interventions alone were also included; and (g) reporting more than one of the following primary or secondary outcomes.

### 2.4. Exclusion Criteria

(a) Nonclinical studies (experimental and basic studies); (b) observational or retrospective studies; (c) lack of sufficient information on baseline or primary or secondary outcome data; (d) narrative reviews, systematic reviews, case reports, letters, editorials, clinical guidelines, and commentaries.

### 2.5. Primary Outcome

Any change in cognitive function (such as MMSE, MoCA, and CDR).

### 2.6. Secondary Outcome

Any change in memory function (such as LMD, DSF, and DSB).

### 2.7. Patient and Public Involvement

Neither patients nor public were involved in the design of this study. This systematic review and meta-analysis did not recruit any patients.

### 2.8. Data Collection

Data were extracted by two independent reviewers (RG and YG) using a standardized form including study demographics, baseline characteristics, study design, intervention methods, outcome measures, and results. We resolved any disagreement through discussion and we consulted a third review author (CZ or XL).

### 2.9. Bias Risk Assessment

According to the risk of bias assessment tool from the Cochrane Handbook for Systematic Reviews of Interventions version 6.0 (updated July 2019), two authors independently assessed the risk of bias of the included study, and any conflicts were resolved through consensus. Bias risk assessment was evaluated from the following seven items: random sequence generation, assignment concealment, blinding of participants and personnel, blinding of outcome assessment, incomplete outcome data, selective reporting, and other bias. These items are described as green, yellow, and red colors and “+”, “−”, and “?”. The symbols indicate “low”, “high”, and “unclear” risk of bias.

### 2.10. Statistical Analysis

The statistical analyses were performed by using Review Manager software (RevMan version 5.3, Cochrane Collaboration, Oxford, UK). Weighted mean difference (WMD) and 95% CI were used as the effect quantity to merge the continuous variables included in the study. *P* value and *I*^2^ statistic were used to test heterogeneity between trial results. When more than two articles were included, heterogeneity was considered. If the *I*^2^ was >50%, the random effect model was applied according to the clinical heterogeneity. Subgroup analysis was used to evaluate the source of heterogeneity. The statistical calculation process was completed by RevMan 5.3 software.

## 3. Results

### 3.1. Literature Search

**Figure 2:**
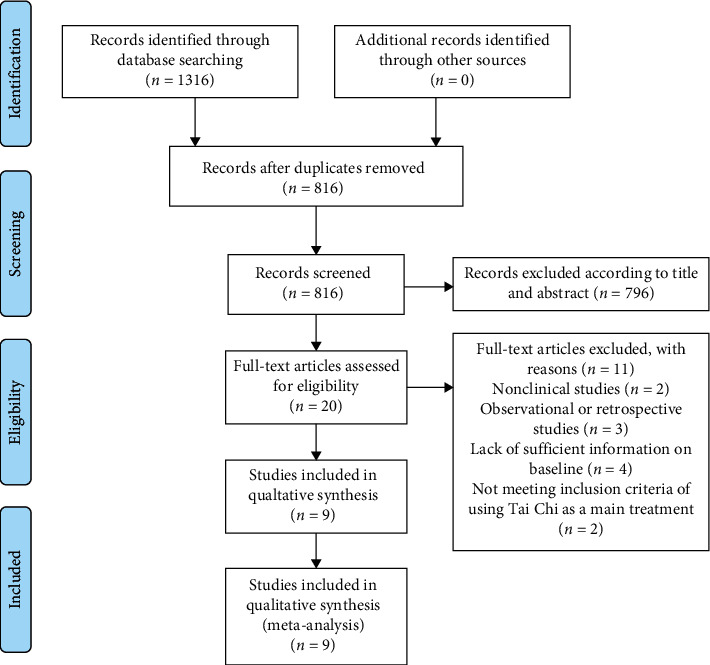
Flowchart of study selection.

Initial searches generated 1316 related literatures. According to the inclusion criteria and exclusion criteria, 9 literatures were included [16, 21, 26–32] (see Figure 2).

### 3.2. Characteristics of the Study

Nine articles [16, 21, 26–32] contained 375 cases in the experimental group and 452 cases in the control group (see Tables 1 and 2).

### 3.3. Risk of Bias

**Figure 3:**
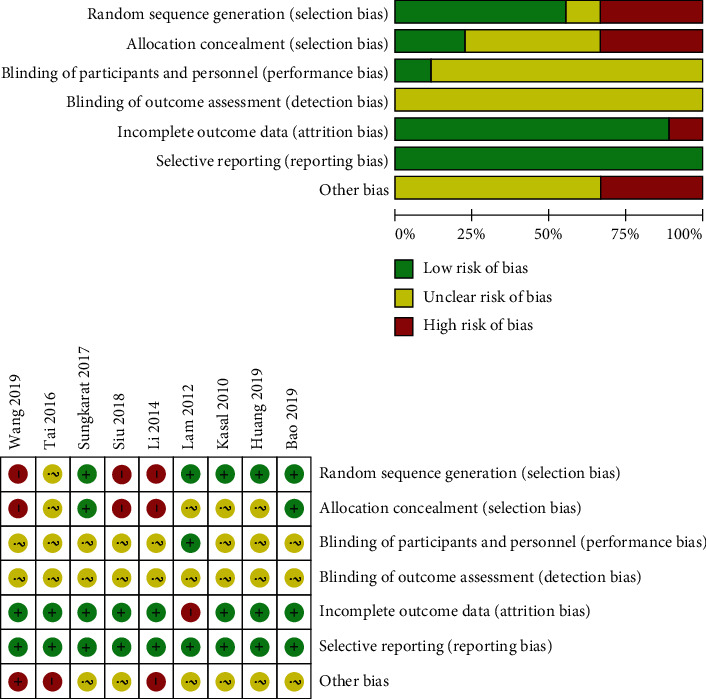
Quality assessment of the included studies.

The results of the risk of bias assessment of the 9 studies [16, 21, 26–32] are summarized in Figure 3. Three literatures [28, 29, 31] were scored as high risk without using random sequence generation. All literatures did not describe detection bias. One article [27] was scored as high risk with incomplete outcome data. All trials measured outcomes listed in their studies and reported on all expected outcome measures of interest (low risk of bias).

### 3.4. Mini-Mental State Examination

**Figure 4:**
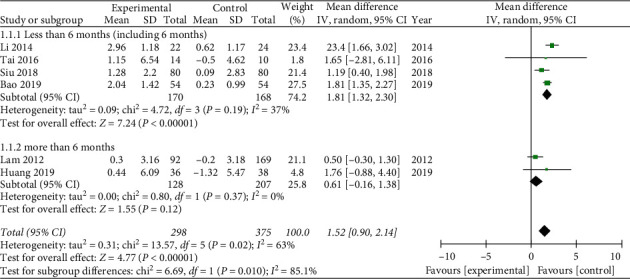
Forest plot of Mini-Mental State Examination.

Six literatures [16, 21, 27, 28, 30, 32] reported the MMSE. Subgroup analysis was carried to analyze heterogeneity. American Academy of Neurology guideline reported that short-term exercise training (6 months) is likely to improve cognitive measure [35]. Therefore, we divide MMSE into different time periods in order to assessing short-term (less than 6 months) and long-term (more than 6 months) effects. In terms of practicing Tai Chi for less than 6 months (including 6 months), the combined effect is WMD = 1.81, 95% CI [1.32, 2.30], *P* < 0.05. The data were statistically significant. On the other hand, the result of practicing Tai Chi for more than 6 months is WMD = 0.61, 95% CI [−0.16, 1.38], *P*=0.12, which was not statistically significant. The total combined effect is WMD = 1.52, 95% CI [0.90, 2.14], *I*^2^ = 63%, and the data were statistically significant. It indicated that practicing Tai Chi less than 6 months (including 6 months) might improve MMSE for patients with cognitive impairment (see Figure 4).

### 3.5. Montreal Cognitive Assessment

**Figure 5:**
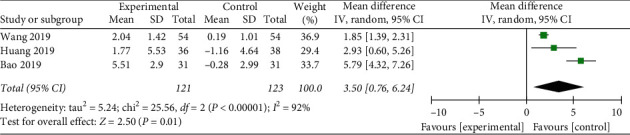
Forest plot of Montreal Cognitive Assessment.

Three literatures described the MoCA [16, 31, 32]. The combined effect was WMD = 3.5, 95% CI [0.76, 6.24], *P* < 0.05. The data was statistically significant (see Figure 5).

### 3.6. Clinical Dementia Rating

**Figure 6:**
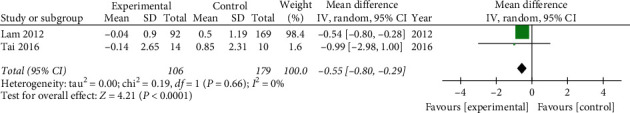
Forest plot of Clinical Dementia Rating.

Two literatures reported CDR [21, 27]. The combined effect was WMD = −0.55, 95% CI [−0.80, −0.29], *P* < 0.05, which was statistically significant (see Figure 6).

### 3.7. Logical Memory Delayed Recall Score

**Figure 7:**
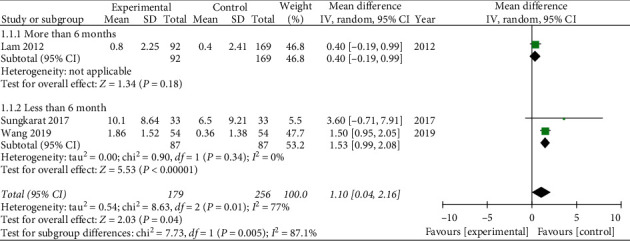
Forest plot of logical memory delayed recall score.

Three literatures reported LMD [27, 29, 31]. One article [27] described practicing Tai Chi for more than 6 months, and the MD = 0.4, 95% CI [−0.19, 0.99], *P*=0.18, which had no statistical significance. In terms of practicing Tai Chi for less than 6 months, WMD = 1.53, 95% CI [0.99, 2.08], *P* < 0.05. The result was statistically significant, and it indicated that practicing Tai Chi less than 6 months might improve LMD (see Figure 7).

### 3.8. Digit Span Forward

**Figure 8:**
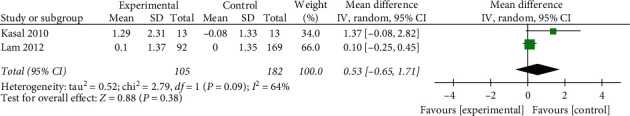
Forest plot of digit span forward.

Two experiments [26, 27] mentioned DSF, and the combined effect amount was WMD = 0.53, 95% CI [−0.65, 1.71], *P*=0.38. It included 105 cases in the experimental group and 182 cases in the control group. The comparison results were not statistically significant and the evidence level was low (see Figure 8).

### 3.9. Digit Span Backward

**Figure 9:**
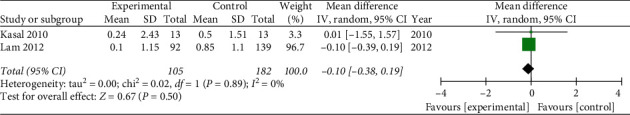
Forest plot of digit span backward.

DSB was included in two articles [26, 27]. The combined effect amount was WMD = −0.1, 95% CI [−0.38, 0.19], *P*=0.5. It had no statistical significance (see Figure 9).

### 3.10. Adverse Events

No article reported the adverse events of Tai Chi.

## 4. Discussion

### 4.1. Summary of Main Findings

The objective of this review was to summarize and evaluate the effectiveness of Tai Chi for cognitive impairment. Nine researches including 827 patients were carried out in China, Brazil, and Thailand. The evidence shows that Tai Chi is more likely to improve cognitive impairment comparing to control group. We found statistically significant benefits of Tai Chi as follows: MMSE WMD = 1.52, 95% CI [0.90, 2.14]; MoCA WMD = 3.5, 95% CI [0.76, 6.24]; CDR WMD = −0.55, 95% CI [−0.80, −0.29]; LMD WMD = 1.1, 95% CI [0.04, 2.16]. However, DSF WMD = 0.53, 95% CI [−0.65, 1.71], and DSB WMD = −0.1, 95% CI [−0.38, 0.19], were not statistically significant. In addition, there is no research reporting the adverse events of Tai Chi and it may be a safe exercise for people with cognitive impairment.

### 4.2. Applicability of the Current Evidence

Previous Cochrane Review showed Tai Chi did significantly reduce risk of falling [4], but it did not analyze any influence on cognitive impairment. It had been affirmed that exercising activities play a positive role in declining risk of the elderly [14, 33], but the effect of Tai Chi was uncertain. Other correlative meta-analyses only focused on English databases [14, 36]. We added studies from Chinese databases in our systematic review. Therefore, this study is an update meta-analysis which evaluates the role of Tai Chi in the prevention of cognitive impairment. Cognitive impairment is a long-term process [2] and it is often considered irreversible [37]. In recent years, performing Tai Chi has been stressed in the process of preventing cognitive impairment [34]. Our research finds that practicing Tai Chi is helpful for cognitive function, but it seems to have no effect on logical memory. Considering that the test results are not necessarily positively correlated with clinical symptoms and conditions, this study only provides reference for clinical practice.

### 4.3. Limitations of This Review

Although new published literatures were added to this systematic review, the risk of this limitation has not been avoided. First, according to Cochrane's bias risk assessment tool, 5 of the 9 included studies are considered to have a high bias risk due to the lack of randomization, blind method implementation, and distribution concealment. The quality of evidence is not good enough because most researches did not describe the detailed methods in their research. Second, the type of Tai Chi was not assessed in this study according to lack of related RCTs. Third, different severity of cognitive impairment may lead to different effects by performing Tai Chi, but no article mentioned that.

### 4.4. Implications for Further Studies

This study provides a certain value for the future research [38]. The current data show that the Tai Chi group has better cognitive function score than the control group and Tai Chi is a safe exercise. In terms of clinical rehabilitation, more long-term follow-ups are needed to provide more RCTs and mechanical researches. In addition, most of the included studies either inadequately reported or did not clearly report methods related to important biases such as randomization/allocation concealment and blinding methods. Future trials should be improved for their reporting quality by following the Consolidated Standards of Reporting Trials (CONSORT) statement [39].

## 5. Conclusion

There is limited evidence supporting that practicing Tai Chi can bring better changes in cognitive function score (MMSE, MoCA, CDR, and LMD). However, there is no influence on DSF and DSB. Current evidence indicates that Tai Chi is a safe exercise for people with cognitive impairment. There is still a need for increasing RCTs to address whether practicing Tai Chi is effective for patients with cognitive impairment.

## Figures and Tables

**Table 1:** Characteristics of the literatures.

Study	Exp. average age/range	Exp. group number	Con. average age/range	Con. group number	Exp. group method	Con. group method	Duration of Tai Chi	Country	Measure	Research designs
Kasal 2010 [26]	73.54	13	74.54	13	Tai Chi	N/A	6 months	Brazil	5.6	RCT
Lam 2012 [27]	77.2	92	78.3	169	Tai Chi + stretching and relaxation exercises	Stretching and relaxation exercises	12 months	China	1.3.4.5.6	RCT
Li 2014 [28]	75	22	77	24	Tai Chi	N/A	14 weeks	China	1	RCT
Tai 2016 [21]	70.21	14	76.3	10	Tai Chi	Nonhealth-related social activities	6 weeks	China	1.3	RCT
Sungkarat 2017 [29]	68.3	33	67.5	33	Tai Chi + education	Education	15 weeks	Thailand	4	RCT
Siu 2018 [30]	—	80	—	80	Tai Chi	N/A	16 weeks	China	1	RCT
Huang 2019 [16]	81.9	36	81.9	38	Tai Chi + routine treatments	Routine treatments	10 months	China	1.2	RCT
Wang 2019 [31]	65–69 (3) 70–79 (32) 80–85 (11)	54	65–69 (2) 70–79 (30) 80–85 (16)	54	Tai Chi	N/A	6 months	China	1.4	RCT
Bao 2019 [32]	65.62	31	68.22	31	Tai Chi + health education	Health education	6 months	China	1.2	RCT

Measure: 1, Mini-Mental State Examination; 2, Montreal Cognitive Assessment; 3, Clinical Dementia Rating; 4, logical memory delayed recall score; 5, digit span forward; 6, digit span backward.

**Table 2:** Characteristics of the literature's inclusion criteria of cognitive impairment.

Study	Inclusion criteria of cognitive impairment	Style of Tai Chi
Kasal 2010 [26]	(i) Memory complaint offered by the patient or by family members over the previous year; (ii) screening score of the Rivermead Behavioral Memory Test lower than 10; (iii) Mini-Mental State Examination (MMSE) within normality, corrected by educational level	Yang style
Lam 2012 [27]	(i) CDR of 0.5 or (ii) neuropsychological criteria for amnestic-mild cognitive impairment (MCI) with subjective cognitive complaints [21]; objective memory impairment with reference to delayed recall of list learning test at greater than or equal to 1.5 SD below education- and age-matched subjects with CDR 0; (iii) no previous regular practice of Tai Chi or other mind-body exercise for more than 6 months	Yang 24-form style
Li 2014 [28]	(i) Having MMSE scores between 20 and 30	N/A
Tai 2016 [21]	(i) Alzheimer with a Clinical Dementia Rating (CDR) score of 0.5–1; (ii) upper limb mobility sufficient to perform requisite finger-pointing tasks, such as flexing and extending the shoulder, elbow, wrist, and fingers	Yang style
Sungkarat 2017 [29]	(i) Petersen's criteria for diagnosing amnestic multiple-domain MCI (a-MCI) had scores of 24 or greater on the Mini-Mental State Examination (MMSE) and less than 26 on the Montreal Cognitive Assessment (MoCA), had adequate memory if cued, and comprehended instructions required for study participation	10-form style
Siu 2018 [30]	(i) The CMMSE screening score ranging from 19 to 28, which was corrected based on educational level (≥18 for illiterate respondents and ≥22 for those having received more than two years of schooling)	Yang style
Huang 2019 [16]	(i) Diagnosed with dementia based on the diagnostic criteria 128 of the Diagnostic and Statistical Manual of Mental 129 Disorders, 4th edition; (ii) a clinical dementia 130 rating score <2	N/A
Wang 2019 [31]	(i) According to the diagnostic criteria set by the National Institute on Aging and the Alzheimer's Association (NIA, AA), the patients were screened as MCI, i.e., subjective cognitive function: the patients who complained or knew about cognitive impairment; (ii) objective cognitive function: according to the Peking Union Medical College, version of the total score of MoCA-p is 25 for the elderly aged 65–79 and 2l–24 for the elderly aged 80–85; (iii) the total score of activities of daily living (ADL) is ≤26, and the complex engineering daily living (ADL) is ≥10	8-form style
Bao 2019 [32]	(i) Having memory decline; (ii) the course of disease was more than 3 months; Global Deterioration Scale (GDS) was 2–3, Clinical Dementia Rating Scale was 0.5, memory test score was below 1.5 standard deviation of age and education matched control group, MMSE score met illiteracy (18–21), primary school culture (21–24), secondary school culture (25–27), and daily life ability score was lower than 26; (iii) memory impairment and other aspects of cognitive function retention	Yang style

## Data Availability

No primary data were used in this article.

## Abbreviations

MMSE: Mini-Mental State Examination
MoCA: Montreal Cognitive Assessment
CDR: Clinical Dementia Rating
LMD: Logical memory delayed recall
DSF: Digit span forward
DSB: Digit span backward
RCT: Randomized controlled trial.

## Supplementary Materials

Supplementary Materials PRISMA 2009 checklist.
